# Physicochemical and Microbiological Stability of Two Oral Solutions of Methadone Hydrochloride 10 mg/mL

**DOI:** 10.3390/molecules27092812

**Published:** 2022-04-28

**Authors:** Elena Alba Álvaro-Alonso, Ma Paz Lorenzo, Andrea Gonzalez-Prieto, Elsa Izquierdo-García, Ismael Escobar-Rodríguez, Antonio Aguilar-Ros

**Affiliations:** 1 Pharmacy Department, Infanta Leonor University Hospital, Av. Gran Vía del Este, 80, 28031 Madrid, Spain; elsa.izquierdo@salud.madrid.org (E.I.-G.); ismael.escobar@salud.madrid.org (I.E.-R.); 2 Center for Metabolomics and Bioanalysis (CEMBIO), Faculty of Pharmacy, Universidad San Pablo-CEU, CEU Universities, Urbanización Montepríncipe, 28660 Boadilla del Monte, Spain; pazloga@ceu.es; 3 Central Laboratory of Madrid UR Salud, Infanta Sofía University Hospital, Paseo de Europa, 34, San Sebastián de los Reyes, 28703 Madrid, Spain; agonzalez@ursalud.com; 4 Faculty of Pharmacy, Universidad San Pablo-CEU, CEU Universities, Urbanización Montepríncipe, 28660 Boadilla del Monte, Spain; aguiros@ceu.es

**Keywords:** methadone hydrochloride, pharmaceutical solutions, drug compounding, high performance liquid chromatography, stability study, microbiology

## Abstract

In this article, we studied physicochemical and microbiological stability and determined the beyond-use date of two oral solutions of methadone in three storage conditions. For this, two oral solutions of methadone (10 mg/mL) were prepared, with and without parabens, as preservatives. They were packed in amber glass vials kept unopened until the day of the test, and in a multi-dose umber glass bottle opened daily. They were stored at 5 ± 3 °C, 25 ± 2 °C and 40 ± 2 °C. pH, clarity, and organoleptic characteristics were obtained. A stability-indicating high-performance liquid chromatography method was used to determine methadone. Microbiological quality was studied and antimicrobial effectiveness testing was also determined following European Pharmacopoeia guidelines. Samples were analyzed at days 0, 7, 14, 21, 28, 42, 56, 70, and 91 in triplicate. After 91 days of storage, pH remained stable at about 6.5–7 in the two solutions, ensuring no risk of methadone precipitation. The organoleptic characteristics remained stable (colorless, odorless, and bitter taste). The absence of particles was confirmed. No differences were found with the use of preservatives. Methadone concentration remained within 95–105% in all samples. No microbial growth was observed. Hence, the two oral methadone solutions were physically and microbiologically stable at 5 ± 3 °C, 25 ± 2 °C, and 40 ± 2 °C for 91 days in closed and opened amber glass bottles.

## 1. Introduction

Methadone was synthesized in the 1940s. It is a pure synthetic opioid agonist with strong affinity and activity at the μ-opioid receptors. It is marginally more potent than morphine and has a longer duration of action, but a lower euphoric effect [1]. Thus, methadone is an alternative to morphine in that it has the same analgesic properties but has milder adverse effects [2].

In 1964, it was first used in clinical rehabilitation programs for opiate addictions, such as those associated with heroin. These programs were developed by a research team at the Rockefeller University of New York [3] and are known as methadone maintenance programs (MMP).

In Spain, heroin use peaked in the 1980s [4]. The first Spanish regulations on the prescription and dispensing of methadone for the treatment of opiate dependence appeared in 1983. However, it was not until 1990 that the prescription criteria were standardized and methadone treatment became widespread. Since then, a series of laws on the regulation and implementation of MMP have been passed and continue to be developed [5].

In the autonomous community of Madrid, resolution 189/2018 [6] was implemented in March 2018, which tasked the Hospital Pharmacy Service (HPS) of the Infanta Leonor University Hospital (ILUH) with supplying methadone to the 27 centers for the Comprehensive Care of Drug Addiction Patients (CDAP) of the Madrid Health Care Service, where MMP for opiate addictions are implemented. The aim of this resolution was to centralize the acquisition, preparation, distribution, and dispensing of methadone by the HPS. This initiative represented a first step in changing the pharmacotherapeutic health care model for the treatment of the patients in the program. To date, between 3000 and 5000 patients are prescribed methadone as an opiate substitute for the treatment of heroin-related addiction disorders.

The methadone solution prepared and supplied by the Pharmacy Service of the ILUH to MMP patients is described in the Spanish National Formulary [7] and is formulated with methadone hydrochloride in the raw material form and purified water at 10 mg/mL (M1). Methadone hydrochloride solution can precipitate at pH greater than 6.5 [8,9]. The solution should also be kept in waterproof and topaz glass containers and protected from light [10,11]. This formulation has assigned a beyond-use date (BUD) of 30 days in refrigerated storage. However, there is no mention or bibliography of any physicochemical or microbiological stability study.

This BUD causes several disadvantages, limiting the organizational capacity in the pharmacy service and in CDAP. A longer BUD would allow optimizing the daily workflow of the pharmacy service, to elaborate sufficiently in advance, as well as improve organizational aspects of the CDAP in the dosing tasks, by increasing the adaptability to the individual dispensing needs of methadone maintained patients [12]. It would even allow the dispensing of methadone for longer periods of time, something that nowadays would be a great advantage due to lockdowns imposed by COVID pandemic.

Furthermore, because the methadone daily prepared is an aqueous solution without preservatives in its formulation, a new compounding of methadone hydrochloride in oral solution was designed and validated in the Hospital Pharmacy Service of ILUH, given the possibility of microbial growth. Its composition included methylparaben and propylparaben as preservatives. The final methadone concentration was also 10 mg/mL (M2).

Due to the large volume of methadone solution to be dispensed (around 3500 L per year), the short BUD assigned, and the need to study the alternative methadone oral solution with preservatives, it became necessary to confirm the BUD of both formulations and even allow to increase them. This is only possible through physicochemical and microbiological stability studies.

In the literature, there are several works that describe stability studies of methadone, but they are either formulations with different concentrations, or with other vehicles in their compositions or even preparations for the intravenous route [10,11,13,14,15,16,17].

Finally, it is important to note that all of these stability studies have been made prior to the publication of the Spanish Good Practice guidelines for the preparation of drugs in hospital pharmacy services by the Ministry of Health in 2014 [18]. They affirm that the assignment of BUD longer than those indicated in the bibliography must be validated with physicochemical and microbiological stability of the non-sterile compounding. The same is described in the United States Pharmacopeia (USP) in its chapter 795 dedicated to the elaboration of non-sterile formulations [19]. These stability studies were also published prior to the resolution 189/2018 [6]. This led to the creation of a new compounding unit in the Pharmacy Service of the Infanta Leonor University Hospital, with different preparation material, raw materials, and packaging material [12] from those that had been used before in the literature and, therefore, this new preparation must be validated by means of a physicochemical and microbiological stability study, taking into account these new working characteristics. All of these aspects justified the need to carry out a stability study.

For all these reasons, and with the aim to confirm the BUD established in the Spanish National Formulary for the methadone hydrochloride oral solution and the new formulation with parabens, we designed and carried out a physicochemical and microbiological stability study in three storage conditions for 91 days, in opened and closed amber glass bottles.

## 2. Material and Methods

### 2.1. Reagents, Reference Standards, and Materials

Methadone hydrochloride was purchased from Esteve Pharmaceuticals S.A. (Barcelona, Spain). Methylparaben and propylparaben, acquired from Fagron Iberica (Terrassa, Spain), were used as preservatives. Purified water was obtained from Grifols laboratory (Barcelona, Spain). All were of Pharmacopoeia grade. In the mobile phase, we used acetonitrile HPLC gradient grade purchased from VWR Prolabo Chemicals (Fontenay-Sous-Bois, France). Phosphoric acid, sodium hydroxide (>99%), hydrochloric acid, and hydrogen peroxide were supplied from Panreac (Barcelona, Spain) and Milli-Q water. All reagents and solvents were of analytical grade.

### 2.2. Standard Operating Procedure (SOP) and Storage Conditions

Methadone oral solutions M1 and M2 were carefully prepared in the Pharmacy Service of the ILUH, one with parabens as preservatives and the other one without parabens, using the following SOP: (1) the required quantity of each ingredient for the total amount to be prepared was calculated; (2) each ingredient was accurately weighed; (3) the methadone hydrochloride was placed to the mortar and triturated until a fine powder was obtained; (4) purified water or preservative water (previously elaborated by dissolving and heating the preservatives in a water bath) was added to the powder and mixed to form a uniform solution; (5) an appropriate amount of water was used to make up the volume in a volumetric flask; (6) the final solution was then packaged in amber glass vials and bottles [10,11] that were previously sterilized.

Considering that each analysis must be done in triplicate, according to International Conference on Harmonization (ICH) guidelines [20], the storage environments and the analysis times, we elaborated 6 L per each oral solution (divided into three batches: 2 L for each storage condition). The composition of solutions M1 and M2 is shown in Table 1.

Solutions were packaged in amber glass vials kept unopened until the day of the test (closed containers), and in a multi-dose umber glass bottle opened daily (opened containers) for carrying out the triplicated physicochemical and microbiological analysis, in three store conditions (refrigeration (5 ± 3 °C), room temperature (25 ± 2 °C), and 40 ± 2 °C) and 9 sample times for 91 days (0, 7, 14, 21, 28, 42, 56, 70, and 91). In total, we prepared 660 samples. 

We carried out the physicochemical stability study at the Center for Metabolomics and Bioanalysis (CEMBIO) of the CEU San Pablo University and the microbiological stability study at the Central Laboratory of Madrid UR Health of the Infanta Sofia University Hospital (ISUH). For the conservation of the samples at 40 ± 2 °C the following chambers were used: chamber Vötsch Industrietechnik VC-0057 (Vötschtechnik, Balingen, Germany) and Binder CB Series 9040-0039 Model CB 210 (Binder, Tuttlingen, Germany) in CEMBIO and ISUH, respectively.

### 2.3. Physicochemical Stability Studies

#### 2.3.1. Equipment and Chromatographic Conditions

HPLC analyses were performed on a qualified and calibrated chromatography system, Agilent-Technologies 1100 series (Madrid, Spain), comprising a quaternary gradient pump, an ultraviolet photodiode array detector (UV-DAD), a 100-vial programmable autosampler, a column oven compartment, an automatic injector, and a software controller.

We used a Waters-XTerra^TM^ RP18 (3.5 μm; 4.6 × 100 mm) column. The column temperature was maintained at 40 °C. The mobile phase consisted of acetonitrile as the organic phase (55%) and sodium phosphate 25 mM (phosphoric acid adjusted to pH = 10 with sodium hydroxide 6.0 M) as the aqueous phase (45%). The flow rate was 1.6 mL/min. The injection volume was 5 μL for each chromatographic analysis. The UV-DAD was set at λ = 254 nm.

#### 2.3.2. Validation of the HPLC Method and Forced-Degradation Studies

The methods and their acceptance criteria were established on the basis of the ICH guidelines Q2 (R1) [21]. 

A new and improved HPLC method to determine methadone hydrochloride in the presence of preservatives was developed and validated prior to this work [22]. Linearity, precision and accuracy were calculated. The curve was constructed from methadone working concentrations of 75–125% (7.5, 9.0, 10.0, 11.0, and 12.5 mg/mL) to assess the linear relationship between the concentration of the analyte and the obtained areas. Detection limit (LOD) and quantification limit (LOQ) were also obtained following EURACHEM recommendations [23]. Forced-degradation studies were also performed in acid, in base, and in oxidation conditions. Peak purity was also calculated.

#### 2.3.3. pH Measurements

Measurements were performed in triplicate using a 744 Metrohm^®^ pH meter (Metrohm Ltd., Herisau, Switzerland) calibrated at pH 4.01 and 7.00 on days 0, 7, 14, 21, 28, 42, 56, 70, and 91.

#### 2.3.4. Organoleptic Characteristics

We inspected the organoleptic characteristics, such as odor, color, flavor, presence of foreign particles or precipitates of the M1 and M2 samples stored at each storage environment. To perform the visual inspection of particles, 3 mL of each formulation were taken and transferred to a transparent test tube fitted with a stopper, inverted, and observed for 5 s against a black background, and 5 s against a white background under a very bright light, according to the Royal Spanish Pharmacopoeia [24].

Measurements were also performed on days 0, 7, 14, 21, 28, 42, 56, 70, and 91.

#### 2.3.5. Data Analysis

Stability was defined as the 90–110% recovery of methadone hydrochloride in both solutions during the 91 days of the study following the guidelines on stability testing [18,19,20,25,26,27].

### 2.4. Microbiological Stability Study

The microbiological quality requirements for non-sterile pharmaceutical preparations, specifically for aqueous liquid oral formulas, according to Royal Spanish Pharmacopoeia and European Pharmacopoeia guidelines [28,29,30,31,32], are: <10^2^ CFU/mL of aerobic bacteria (TAMC), <10¹ CFU/mL of yeast and mold (TYMC), and the absence of *Escherichia coli.* If the oral compounding includes preservatives, antimicrobial effectiveness testing must be previously demonstrated [33].

The following materials were used for the tests: 0.9% saline serum, ATCC reference strains of *Staphylococcus aureus* (ATCC 43300), Bacteroides thetaiotaomicrom (ATCC 29741), *Escherichia coli* (ATCC 25922), *Pseudomonas aeruginosa* (ATCC 9027), and *Candida parapsilopsis* (ATCC 2019). They were obtained from Thermo Fisher Scientific (Waltham, MA, USA). The culture media used were MacConkey Agar, CNA agar (nalidixic acid and colistin) with 5% lamb blood, Schaedler agar with vitamin K, and 5% lamb blood and Sabouraud agar with chloramphenicol and gentamicin. They were supplied from Becton Dickinson (Franklin Lakes, NJ, USA). We had the MALDI-TOF technique for the identification of microorganisms [34,35].

Samples of opened bottles were opened every day for a few seconds in the HPS to simulate typical drug dosage. Then, the opened and closed triplicated M1 and M2 samples were sent from the ILUH Pharmacy Service to the ISUH on Tuesdays and were analyzed on days 0, 7, 14, 21, 28, 42, 56, 70, and 91.

Every day of the analysis, 1 mL of the samples was sown in MacConkey Agar and CNA for 48 h, in Schaedler agar in anaerobiosis for 72 h, and in Sabouraud agar for 5 days. TAMC and TYMC were calculated and detection of *Escherichia coli* was performed. For M2 samples, prior to sowing, it was diluted 1:10 with saline to neutralize the action of the preservatives. 

For antimicrobial effectiveness testing, several containers of M2 were seeded with a suspension of one of the test organisms to give an inoculum of 10^5^–10^6^ microorganisms/mL. The volume of the suspension of inoculum did not exceed 1% of the volume of the product. They were incubated at 20–25 °C and followed for 28 days to observe the logarithmic reduction according to Royal Spanish and European Pharmacopoeia.

Finally, tests for specified microorganisms were also performed in accordance with Royal Spanish and European Pharmacopoeia [28,31,32]. The product would comply with the test if no colonies of the specified microorganisms were detected.

## 3. Results

### 3.1. Physicochemical Stability

#### 3.1.1. HPLC Analysis and Forced-Degradation Studies

**Figure 1:**
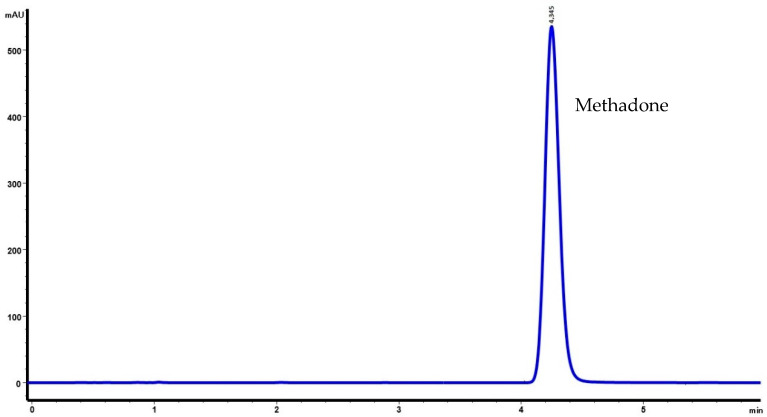
Chromatogram of methadone hydrochloride (10 mg/mL) without preservatives (M1). Retention time for methadone was 4.345 min.

**Figure 2:**
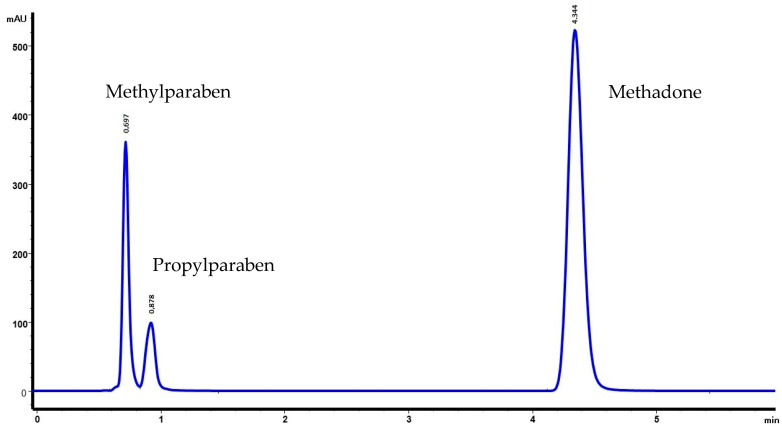
Chromatogram of methadone hydrochloride (10 mg/mL) with preservatives (M2). Retention times for methadone, methylparaben, and propylparaben were 4.344, 0.697, and 0.878 min.

Retention times for methadone, methylparaben, and propylparaben were 4.34, 0.70, and 0.88 min, respectively. The chromatograms obtained are shown in Figure 1 and Figure 2.

The method was linear (y = 284.3x − 97.8, r = 0.996), with y being the obtained areas and x the concentration (mg/mL). Instrumental precision was 0.33% for standards (n = 10); intra-assay precision 0.53% (n = 6), and inter-assay precision 1.95% (n = 12). The relative standard deviation percentage for accuracy was 1.28%. The recovery percentage was 101.5 ± 1.5%.

We calculated that LOQ was 2.18 μg/mL. The LOD is the value capable of detecting the analyte. However, in the 0.0002 mg/mL concentration, four of the six samples were not detected, so the LOD was considered to be the previous concentration value in which methadone was detected. Therefore, LOD was 2.0 μg/mL.

The method was stability-indicating, with a complete separation of the degradation products from the peak of the methadone (in all situations, the methadone peak continued to be obtained at minute four). The most destabilizing conditions were oxidizing and alkaline. The methadone peak purity was 999.830 over 1000.

The percentage of recovery for all samples fulfilled the requirements of the compounding stability studies (90–110%) [26] because there was no significative degradation of methadone hydrochloride throughout the study in all batches in the three storage conditions (Table 2). This shows the stability of all samples throughout the 91 days of analysis. No differences were found between the samples of the closed and opened bottles.

#### 3.1.2. pH Measurements

**Figure 3:**
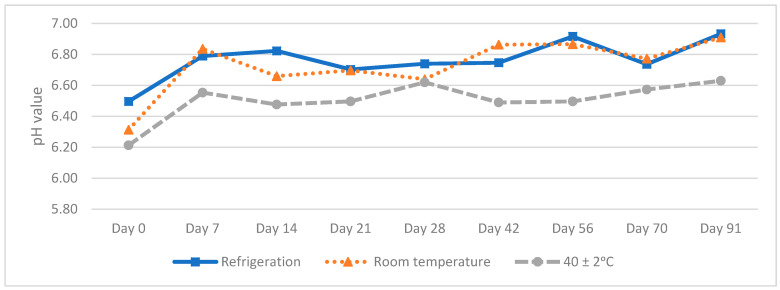
pH values of methadone hydrochloride (10 mg/mL) without the preservative (M1) oral solution.

**Figure 4:**
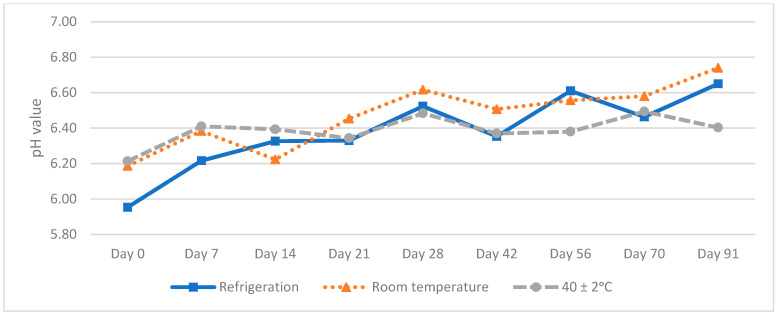
pH values of methadone hydrochloride (10 mg/mL) with the preservative (M2) oral solution.

pH values increased throughout the 91 days of the study. We emphasize that lower values were obtained in the oral solution that contained the preservatives (M2). However, neither of the two solutions reached pH = 7 (Figure 3 and Figure 4). This results ensured no risk of methadone precipitation.

#### 3.1.3. Organoleptic Characteristics

Throughout the study, the samples of both solutions remained transparent, without coloration. No suspended particles or precipitates were observed. They showed no odor. However, the bitter taste (typical of methadone) on day 0 was maintained until the last day of the study. No differences were found in any of the three storage environments.

### 3.2. Microbiological Stability

The antimicrobial efficacy of the preservatives used in the M2 formulation was demonstrated prior to the microbiological stability study, as well as the fertility test of the culture media and the suitability of the counting method in the presence of the product.

Throughout the study, no microbial growth was observed in any sample analyzed (on day 70 it could not be cultured due to the SARS-CoV-2 lockdown). Despite this, the analysis on day 91 did not show any microbial growth either. Therefore, all samples from closed and opened bottles met the microbiological quality acceptance criteria for liquid oral formulas until the end time in the three storage environments.

## 4. Discussion

In this study, we carried out a physicochemical and microbiological stability study of two oral solutions of methadone hydrochloride (10 mg/mL) with and without preservatives, with an increased BUD until 91 days compared to the formulation described in the Spanish National Formulary. 

The stability of methadone hydrochloride has already been studied in the literature. These previous studies have allowed us to avoid sampling in unstable conditions e.g., unprotected from light [11,16] or in plastic packaging material [10], where there was a greater loss of methadone. This fact led us to carry out our stability study in amber glass bottles.

Moreover, those stability studies were carried out on different pharmaceutical preparations of methadone compared to those used in this work. These preparations were elaborated at different concentrations, with other vehicles for oral administration different from water (such us Tang or simple syrup), for administration routes other than the oral route, or using methadone hydrochloride different from the raw material (tablets, injectables, etc.) [10,11,15,16,17]. We also find stability studies of formulations in which methadone was formulated with other drugs [13,36].

The use of preservatives with methadone was described in the literature for the first time by Beaseley and Ziegler [37] in 1977; they observed that the standard methadone solution was unstable and adding 1 mg of sodium benzoate per milliliter to the solutions can stabilize them for at least one month.

Due to the increased use of methylparaben as preservative in the 1980s, Ching et al. [14] carried out a stability study of methadone 5 mg/mL, demonstrating a stability of 4 months but without microbiological data. None of the previously described stability studies used in their composition the two parabens that we used in this work in the M2 solution. We used methylparaben and propylparaben since they are the most widely used preservatives in aqueous solutions formulated, such as preservative water, according to the Spanish National Formulary [38].

The HPLC method developed in our previous study was a rapid, simple, reliable, and economical technique analytically validated and has allowed for the efficient quantification of methadone hydrochloride in an oral solution without interference from preservatives and a concentration and composition not previously analyzed in the literature. This procedure was a new and an improved method in comparison to those described in the United States Pharmacopoeia (USP) [39] and the literature [37,40,41,42,43].

In our stability study, all samples comply with the recommendations. The two formulations (with and without preservatives) remained stable throughout the 91 days of study stored under refrigeration (5 ± 3 °C), room temperature (25 ± 2 °C) and 40 ± 2 °C, indicating that the presence of parabens does not alter the stability of methadone hydrochloride. With regard to pH, we want to highlight that, although the recommendations indicate that methadone hydrochloride should have a value lower than 6.5, in our study we demonstrated that this value can be extended (near to 7) because methadone hydrochloride remained stable at values close to that figure.

Regarding the small pH variations found in both methadone solutions over time, although they were relevant due to the results of our stability study, we can affirm that they were not due to microbial growth or the appearance of degradation products. Therefore, the variations found may be due to the small variations in temperature since the samples were removed from the chamber until their analyses with the pH meter, to the variations of the pH meter, due to the fact that they were not designed to operate in a solution that contained almost no ions.

Regarding the microbiology stability, it is important to highlight that aqueous solutions or suspensions are more unstable and much more vulnerable to microbiological contamination than solid forms. Most of the stability studies, being non-sterile pharmaceutical forms, do not include microbiological stability studies. Furthermore, contamination can reduce or inactivate the therapeutic action of the drug or even form toxic products affecting patient safety. Even the ingestion of pathogenic microorganisms can cause a serious infection in the pediatric population or in the immunosuppressed patient.

With these premises, it was necessary to carry out a microbiological study in which there was no microbial growth in the closed bottles or in those that were opened daily throughout the 91 days of the study, thus demonstrating that both solutions were microbiologically and physicochemically stable without toxic products. 

After our results, unlike the results by Lauriault et al. [15], the addition of preservatives was not necessary to prevent microbial growth, since in our samples, both in the presence or absence of preservatives, no microbial growth was observed after 91 days of study, including those that were kept at room temperature and at 40 ± 2 °C. Furthermore, following the Good Practice Guidelines, standardizing procedures, cleaning, and evaluating the elaborating staff, the risk was minimized.

The evaluation of these two oral solutions has not been published before. Furthermore, a formulation of methadone hydrochloride solution with methylparaben and propylparaben in its composition has never been evaluated before. This fact allowed us to study the stability of an alternative methadone solution, in case the preservative-free solution daily prepared at the Hospital Pharmacy Service is not stable; we also analyzed whether the addition of preservatives, beyond increasing the microbiological quality, could alter the physicochemical stability of the solution.

Moreover, among other advantages of our study, we designed the stability study following literature recommendations and guidelines [20,25,26,27], choosing more sampling times to have more data in case we did not find stability throughout the study. We also chose three storage environments to obtain more information on the stability of methadone in a solution out of the fridge, both at room temperature or 40 ± 2 °C, situations that could arise in case of improper conservation or hot weather. These results offer efficient and necessary information that could be applied, day-to-day, at the Hospital Pharmacy Service, CDAP, and in a patient’s home.

We also want to highlight the logistical and organizational complexity of this stability study, which involved a total of 660 samples (with difficulty in treating and identifying each sample) and three centers that had to be constantly coordinated.

After this study, the physicochemical and microbiological stability, the classical formulation (M1), and the new alternative solution (M2) were demonstrated. Thanks to our study, the main advantage and practical application is that we increased the beyond-use date of the methadone solution, which made it possible to improve the workflow organization in the daily preparations of the Hospital Pharmacy Service. More importantly, it also allows for better organization in centers, especially during holiday periods or due to a lack of staff. It even allows adapting the dispensing to the needs of patients receiving methadone treatment. We hope to publish these results (that we are measuring) in the future.

## 5. Conclusions

Two oral methadone hydrochloride solutions (10 mg/mL), with and without parabens as preservatives, were physically and microbiologically stable at 5 ± 3 °C, 25 ± 2 °C, and 40 ± 2 °C, for 91 days in previously sterilized closed and opened bottles.

The results obtained after this stability study allowed to increase the BUD of the methadone hydrochloride oral solution prepared daily at the Hospital Pharmacy Service of the Infanta Leonor University Hospital for the patients under methadone maintenance programs in the community of Madrid.

## Figures and Tables

**Table 1:** Composition of methadone hydrochloride (10 mg/mL) oral solutions.

	M1 (g)	M2 (g)
Methadone hydrochloride	20	20
Methylparaben	-	1
Propylparaben	-	0.44
Purified water to	2 L	2 L

M1: methadone hydrochloride oral solution (10 mg/mL) without parabens; M2: methadone hydrochloride oral solution (10 mg/mL) with parabens.

**Table 2:** Percentage of recovery of both methadone hydrochloride oral solutions.

			Day 0	Day 7	Day 14	Day 21	Day 28	Day 42	Day 56	Day 70	Day 91
M1	Closed	Refrigeration	99.56 ± 0.26	99.92 ± 0.05	99.21 ± 0.14	99.86 ± 0.36	99.99 ± 0.19	99.61 ± 0.18	99.00 ± 0.20	100.89 ± 0.20	99.26 ± 0.08
		Room temperature	100.6 ± 0.41	101.17 ± 0.10	100.30 ± 0.14	100.09 ± 0.28	100.80 ± 0.21	100.25 ± 0.49	99.67 ± 0.51	101.88 ± 0.38	100.19 ± 0.29
		40 ± 2°C	100.42 ± 0.20	100.28 ± 0.28	99.81 ± 0.12	100.16 ± 0.26	100.38 ± 0.32	99.99 ± 0.33	99.50 ± 0.09	101.64 ± 0.44	99.94 ± 0.63
	Opened	Refrigeration	100.28 ± 0.13	100.14 ± 0.52	99.26 ± 0.48	100.40 ± 0.44	99.89 ± 0.32	99.77 ± 0.39	99.51 ± 0.17	101.04 ± 0.29	99.27 ± 0.56
		Room temperature	104.29 ± 3.34	100.12 ± 0.05	99.36 ± 0.32	100.45 ± 0.32	99.71 ± 0.12	100.06 ± 0.30	99.84 ± 0.24	100.59 ± 0.40	99.76 ± 0.11
		40 ± 2 °C	99.72 ± 0.21	100.08 ± 0.17	99.19 ± 0.31	100.12 ± 0.05	99.69 ± 0.19	99.34 ± 0.31	99.43 ± 0.32	101.56 ± 0.24	99.62 ± 0.69
M2	Closed	Refrigeration	100.54 ± 0.10	100.43 ± 0.31	99.77 ± 0.09	100.39 ± 0.01	100.16 ± 0.06	99.82 ± 0.01	99.91 ± 0.02	100.70 ± 0.11	101.14 ± 0.02
		Room temperature	100.61 ± 0.37	101.01 ± 0.14	100.13 ± 0.02	100.69 ± 0.03	100.26 ± 0.04	100.63 ± 0.03	100.01 ± 0.05	101.65 ± 0.02	101.34 ± 0.04
		40 ± 2 °C	100.75 ± 0.10	100.44 ± 0.17	99.81 ± 0.01	100.37 ± 0.01	100.27 ± 0.05	100.30 ± 0.01	100.12 ± 0.03	101.94 ± 0.03	100.75 ± 0.07
	Opened	Refrigeration	99.79 ± 0.26	100.10 ± 0.23	99.29 ± 0.07	100.00 ± 0.01	99.48 ± 0.06	99.82 ± 0.01	99.71 ± 0.04	101.61 ± 0.03	99.31 ± 0.01
		Room temperature	100.07 ± 0.35	100.45 ± 0.17	99.15 ± 0.03	99.95 ± 0.02	99.83 ± 0.01	100.14 ± 0.02	99.79 ± 0.05	101.18 ± 0.04	99.62 ± 0.02
		40 ± 2 °C	99.76 ± 0.20	100.03 ± 0.20	99.19 ± 0.03	99.97 ± 0.06	99.79 ± 0.03	99.41 ± 0.02	99.94 ± 0.02	101.46 ± 0.05	99.76 ± 0.03

M1: methadone hydrochloride oral solution (10 mg/mL) without parabens; M2: methadone hydrochloride oral solution (10 mg/mL) with parabens.

## Data Availability

The datasets used and/or analyzed in the current study are available from the corresponding author upon reasonable request.
